# Effects of music therapy on degree of cooperation with anesthesia induction and preoperative anxiety in children with simple congenital heart disease: A protocol of systematic review and meta-analysis

**DOI:** 10.1371/journal.pone.0296287

**Published:** 2023-12-27

**Authors:** Haoyu Liu, Xiaojin Song, Lu Xiong, Liyun Zhang, Bingquan Luo, Siling Liu

**Affiliations:** 1 Postgraduate Department, Capital University of Physical Education and Sports, Haidian, Beijing, China; 2 Postgraduate Department, Beijing Sport University, Haidian, Beijing, China; 3 Department of Public Sports, Jiangxi Institute of Applied Science and Technology, Nanchang, Jiangxi, China; 4 Taiyuan Tong Shan Rehabilitation Hospital, Taiyuan, Shanxi, China; 5 School of Sport and Art, Shenzhen Technology University, Shenzhen, Guangdong province, China; Jinnah Sindh Medical University, PAKISTAN

## Abstract

**Background:**

Anxiety is a common preoperative symptom in children with simple congenital heart disease (SCHD). Music therapy shows potential as a non-drug intervention. However, it is unclear how it impacts the level of cooperation during the induction of anesthesia and preoperative anxiety, as well as the factors that influence its effectiveness. Therefore, we will conduct a comprehensive review and meta-analysis to assess the impact of music therapy on the level of cooperation during anesthesia induction and preoperative anxiety in children with SCHD.

**Methods:**

Electronic searches will be conducted through various databases including PubMed, Embase, Web of Science, Medline, and CNKI to gather randomized controlled trials (RCTs) examining the impact of music therapy on the level of cooperation during anesthesia induction and preoperative anxiety among children with SCHD. Two evaluators will independently review the literature, extract information, and assess the risk of bias in the included studies. Afterwards, data analysis will be conducted using Stata 14.0 software and Revman 5.4 software. The results will be based on random-effects models. The reliability and quality of evidence will be evaluated by using the Grading of Recommendations, Development, and Evaluation (GRADE) system. Heterogeneity will be examined by subgroup analysis stratified by age, gender ratio, type of surgery, drop-out rate, measurement tools, and country of origin. We will assess potential publication bias using funnel plot symmetrical and Begg’s ang Egger’s regression tests.

**Discussion:**

Given the multiple advantages that may be associated with music therapy, this therapy may be a desirable alternative to existing therapies for preoperative cooperation and anxiety issues in children with SCHD. We hope that this systematic review will guide clinical decision-making for future efforts related to coping with preoperative fit and anxiety in children with SCHD.

**Systematic review registration:**

PROSPERO registration number: CRD42023445313. https://www.crd.york.ac.uk/prospero/display_record.php?ID=CRD42023445313.

## Introduction

Simple Congenital Heart Disease (SCHD) is characterized by significant structural abnormalities in the heart or intrathoracic great vessels [1]. It encompasses conditions such as atrial septal defect, ventricular septal defect, and mild pulmonary stenosis [2]. The main symptoms associated with SCHD include cyanosis [3], shortness of breath, fatigue, heart murmurs [4], poor weight gain or growth, and recurrent respiratory infections [5]. Surgical treatment of children with SCHD is associated with a significant incidence of preoperative anxiety, ranging from 40% to 60% during the preoperative period [6]. High levels of anxiety not only decrease the child’s level of cooperation during the induction of anesthesia but also increase the risk of post-surgery complications such as delirium and behavioral changes [7]. Furthermore, this anxiety can contribute to increased restlessness following general anesthesia and the development of maladaptive behaviors, such as difficulty falling asleep, nocturnal enuresis, fear of darkness, and resistance at bedtime [8].

Various measures are frequently employed to alleviate preoperative anxiety in children, such as clown intervention [9], distraction techniques [10], quasi-premedication [11], behavioral preparation programs [12], and virtual reality [13,14]. Nevertheless, it should be noted that all of these approaches are associated with certain limitations.

Music therapy is an effective approach for supporting children with anxiety, in which the therapist utilizes music as the primary tool to establish a connection and engage with the patient. This therapy has demonstrated efficacy in treating children and adolescents with anxiety disorders [15,16]. Musical intervention is an increasingly utilized therapeutic resource in nursing care as a complementary therapy to promote relaxation, emotional and spiritual comfort, distraction, a sense of wellness, and pain relief in hospitalized patients [17]. Music reduces anxiety by modulating the autonomic nervous system, which controls crucial bodily functions, including heart rate, digestion, respiration rate, and pupillary response [18,19]. Several studies have shown that music listening reduces cortisol levels and other neuropeptides related to the hypothalamic-pituitary-adrenal axis [20–22]. This mechanism activates the limbic system, releasing endorphins, which reduce discomfort and pain while maximizing pleasure [23,24]. However, various individual factors, such as age, gender, emotional condition, music preference, personal connections with the music, musical education, and cultural background, may influence the effectiveness of music therapy interventions. To improve the precise application of music therapy in reducing preoperative anxiety and promoting cooperation during anesthesia induction in children with SCHD, it is essential to conduct a comprehensive review.

## Methods

### Protocol and registration

The study has been registered with PROSPERO (registration number: CRD42023445313). To achieve the systematic review protocol, we will follow the Preferred Reporting Items for Systematic Reviews and Meta-analysis Protocol guidelines [25]. This research involves collecting and examining primary data; therefore, ethical approval is not required.

### Data sources and search strategies

We will perform an extensive literature search utilizing five electronic databases: Web of Science, Embase, PubMed, Medline, and China National Knowledge Infrastructure (CNKI). We will search for articles published from the commencement of each respective database until September 2023. Two review team members (HL and SL) formulated comprehensive search strategies for each electronic database. A topic search was conducted in English-language databases using the following relevant terms: preoperative, child*, music, and random*. We plan to use equivalent search terms in the Chinese-language databases. Search strategies are shown in Table 1. We will import these search results into Endnote X9 software (Thomson Reuters, Windows version) and check for duplicates. After removing them accordingly, we will remove the duplicate literature that was not identified by comparing authors, titles and publication dates.

**Table 1:** Search strategy.

Databases	Search strategies
Web of science	(((TS = (preoperative)) AND TS = (child*)) AND TS = (music)) AND TS = (random*)
**Embase**	preoperative:ab,ti AND child*:ab,ti AND music:ab,ti AND random*:ab,ti
**Medline**	AB preoperative AND AB child* AND AB music AND AB random*
**Pubmed**	(((preoperative[Title/Abstract]) AND (child*[Title/Abstract])) AND (music[Title/Abstract])) AND (random*[Title/Abstract])
**CNKI**	**SU = ’音乐’ and SU = ’儿童’ and SU = ’随机’ and SU = ’术前’**

### Eligibility criteria

#### Type of study

This study aims to conduct a systematic review and meta-analysis of randomized controlled trials (RCTs) to examine the impact of music therapy on the level of cooperation during anesthesia induction and preoperative anxiety in children with SCHD.

#### Types of participants

(1) Diagnosed with SCHD (atrial septal defect, ventricular septal defect, or patent ductus arteriosus) through transthoracic echocardiography; (2) age between 0 and 12 years; (3) first operation; (4) normal mental, psychological, and intellectual development; and (5) absence of evident hearing difficulties.

#### Interventions and comparators

The control group received standard SCHD care, including primary care, intensive care, expert care, and health instruction. Meanwhile, the experimental group received music therapy and routine SCHD care.

#### Types of outcome measures

Degree of cooperation with anesthesia induction and preoperative anxiety.

#### Language restriction

No linguistic or date restriction will be applied as part of the eligibility criteria.

### Study selection and data extraction

**Fig 1:**
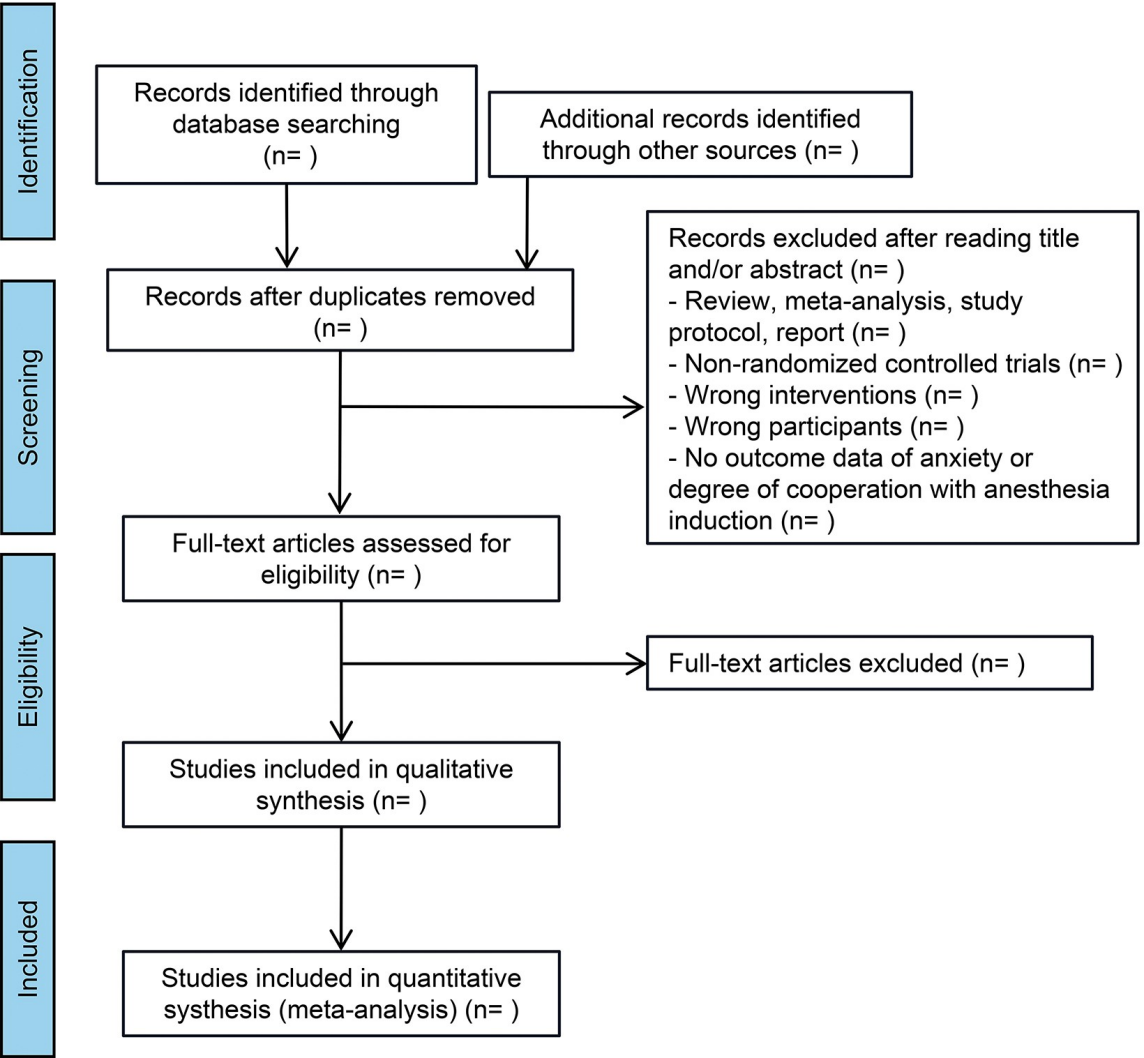
Flowchart steps of the systematic review.

Two reviewers (HL and SL) will independently evaluate the eligibility of articles by thoroughly reviewing their titles, abstracts, and complete texts according to the predefined criteria for inclusion and exclusion (Fig 1). Any differences between the two reviewers will be resolved through discussion and consultation with a third reviewer (BL). Relevant data, such as the author’s name, publication year, country, type of surgery, participant count, gender distribution, dropout rate, age, intervention techniques, duration of intervention, measurement instruments, and average and standard deviation of continuous outcome measures, will be extracted from the analyzed articles.

### Methodological quality assessment

The quality of the selected studies will be assessed independently by two researchers (HL and SL) using the Cochrane Collaboration tool for RCTs [26]. The items are assessed using three categories: low risk of bias, unclear bias, and high risk of bias. We will assess the following characteristics: random sequence generation (selection bias), allocation concealment (selection bias), blinding of participants and personnel (performance bias), incomplete outcome data (attrition bias), selective reporting (reporting bias), and any additional biases resulting from these factors will be graphed and evaluated using Review Manager 5.4. Discrepancies will be resolved through discussions among the authors or with the involvement of a third member (LX), as required.

### Grading of evidence

The Grading of Recommendations Assessment, Development, and Evaluation (GRADE) method will be utilized to evaluate the quality of evidence for each outcome [27]. Evidence will be divided into four categories: high, medium, low, and very low.

### Statistical analysis

#### Additions of missing data

If an article lacks or has insufficient data, basic information will be emailed to the relevant authors. Studies will be excluded if contact is not established or the data is insufficient.

#### Measures of treatment effect

A meta-analysis will be performed utilizing Revman 5.4 software, which the Cochrane Collaboration created. For continuous variables with different measurements, we will utilize the standard mean difference (weighted by the inverse of the variance). We will display 95% confidence intervals (CIs) as forest plots in both cases. Statistical significance was set at p-values < 0.05. The Chi-square test will be employed to evaluate the heterogeneity of the included RCTs. An I^2^ value of 50% or higher and p < 0.10 indicated the presence of heterogeneity. If I^2^ ≤ 50% and p ≥ 0.10, a fixed-effects model will be used for analysis; Otherwise, a random-effects model will be employed. To further analyze the influence of heterogeneity on the conclusions of the meta-analyses, subgroup analyses were conducted.

If a quantitative synthesis is deemed inappropriate, the findings of each study will be summarized and discussed, taking into account the risk of bias and the significance of the results. Subsequently, interventions that demonstrate effectiveness and offer potential conclusions for future studies and informed decision-making will be identified after consolidating the results.

#### Sensitivity analysis

To conduct the sensitivity analysis, we will identify the sources of heterogeneity by re-estimating the combined effects using the one-by-one elimination method.

#### Subgroup analysis

Subgroup analyses will be performed based on age, gender ratio, type of surgery, drop-out rate, measurement tools, and country of origin if there is notable variation in the study data.

Age-related psychological characteristics, gender differences in anxiety, invasive degree of surgery, sample error, validity differences of measurement tools, and cultural environment of subjects are all likely to affect the final test results.

#### Publication bias analysis

To assess potential publication bias, funnel plots will be created, with a requirement of including a minimum of 10 articles [28]. Funnel plots will be visually examined for asymmetry, and Egger’s test will be employed to conduct sensitivity analysis and statistically evaluate publication bias.

### Amendments

The information will be presented in the final report, along with any modifications to the protocol.

## Discussion

Preoperative anxiety significantly affects the surgical cooperation and postoperative rehabilitation of children with uncomplicated chronic heart disease. Children with SCHD must be admitted to the operating room alone and anesthetized before surgery. Music therapy is characterized by its low cost, lack of side effects, and wide applicability [29,30]. The interventions have been shown to regulate emotions [31], distract attention [32], and support personalized treatment [33]. To the best of our knowledge, no previous systematic review and meta-analysis have been performed to investigate the effects of music therapy on children with congenital heart disease. Moreover, several individual factors, such as age, gender ratio, type of surgery, drop-out rate, measurement tools, and country of study, will likely impact the results. Our systematic review and meta-analysis can provide valuable insights for the clinical management and enhancement of the psychological status in SCHD children.

Currently, the efficacy and safety of music therapy in improving degree of cooperation with anesthesia induction and preoperative anxiety in SCHD children have been confirmed by several RCTs [34–36], while none relevant systematic evaluation studies exist. This will be the first systematic review and meta-analysis protocol to assess the efficacy and safety of music therapy in SCHD children’s cooperation with anesthesia induction and preoperative anxiety. In this systematic evaluation, a detailed summary of the up-to-date evidence relevant to music therapy for children with simple congenital heart disease will be provided. This evidence will be helpful to the specific use of music therapy for SCHD children’s surgery.

This study has some limitations. Primarily, our focus will be solely on the scrutiny of published studies, discluding any unpublished works from evaluation. In addition, because the number and insufficient quality of existing RCTs of SCHD children in improving degree of cooperation with anesthesia induction and preoperative anxiety are limited, we need further high-quality RCTs to consolidate the clinical evidence supporting music therapy as a potential treatment option for improving cooperation with anesthesia induction and preoperative anxiety.

## Supporting information

S1 Checklist PRISMA-P 2020 checklist. (DOC)
